# Dynamics in Pro‐Inflammatory Cytokines in Response to Hypoglossal Nerve Stimulation in Obstructive Sleep Apnea

**DOI:** 10.1002/lio2.70472

**Published:** 2026-08-15

**Authors:** Ralph Pries, Yi Cai, Kirstin Plötze‐Martin, Sanja Jelic, Jonas Fleckner, Karl‐Ludwig Bruchhage, Armin Steffen

**Affiliations:** ^1^ Department of Otorhinolaryngology‐Head and Neck Surgery University of Luebeck Lübeck Germany; ^2^ Department of Otolaryngology‐Head and Neck Surgery Columbia University New York New York USA; ^3^ Department of Medicine Columbia University New York New York USA

**Keywords:** cytokines, hypoglossal nerve stimulation therapy, inflammation, obesity, obstructive sleep apnea, sleep apnea surgery, upper airway stimulation

## Abstract

**Objective:**

Obstructive sleep apnea (OSA) is associated with chronic low‐grade systemic inflammation. In cases of continuous positive airway pressure (PAP) failure, hypoglossal nerve stimulation (HNS) is a treatment option for OSA but its impact on systemic inflammation to date is unknown. Herein, we investigated changes in cytokines and endothelial markers before and after HNS.

**Methods:**

Eighteen adults with moderate or severe OSA underwent fasting blood sample collection before and 6–8 months after HNS implantation. Plasma levels of interleukin‐6 (IL‐6), tumor necrosis factor‐α (TNF‐α), vascular endothelial growth factor (VEGF), angiopoietin‐2 (Ang2), C‐reactive protein (CRP), and E‐selectin were measured using ELISA. Clinical metrics, including apnea‐hypopnea index (AHI) and Epworth Sleepiness Scale (ESS), were assessed.

**Results:**

HNS significantly reduced AHI (from 25.9 ± 13.4 to 14.2 ± 10.5) and ESS scores (from 12.6 ± 5.0 to 7.1 ± 4.0). There were significant decreases in VEGF (*p* = 0.033) and TNF‐α (*p* = 0.0013) with HNS. CRP levels were elevated among OSA patients when compared with healthy donors and did not change after HNS. IL‐6 levels did not change with HNS, but were low in both healthy and OSA patients. No significant overall changes were observed in Ang2 or E‐selectin. Obese participants exhibited higher baseline inflammatory profiles compared with non‐obese participants.

**Conclusions:**

In this small cohort, HNS improved OSA severity and was associated with decreases in VEGF and TNF‐α while other inflammatory markers were unchanged. Larger, long‐term studies are needed to understand the potential of HNS to confer cardiometabolic benefits through reduction of systemic inflammation in OSA.

## Graphical abstract

Obstructive sleep apnea (OSA) is associated with chronic low‐grade systemic inflammation. In cases of continuous positive airway pressure (PAP) failure, hypoglossal nerve stimulation (HNS) is a treatment option for OSA but its impact on systemic inflammation to date is unknown. Our study indicates that HNS improves OSA severity and is associated with decreases in VEGF and TNF‐α, suggesting the mitigation of systemic inflammation with HNS.

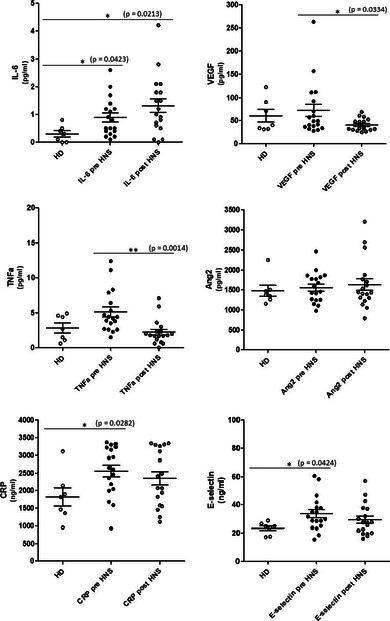

## 1 Introduction

Obstructive sleep apnea (OSA) is a common sleep disorder and a major risk factor for cardiovascular and metabolic diseases [1, 2]. OSA is characterized by *recurrent nocturnal breathing interruptions*, leading to a reduction (hypopnea) or total cessation (apnea) of airflow in the upper airways accompanied by intermittent hypoxia [3]. The current standard treatment for OSA is positive airway pressure (PAP) therapy, whereas hypoglossal nerve stimulation (HNS) has been established as a promising second‐line treatment option for patients with PAP failure [4, 5]. OSA‐driven oxidative stress is closely associated with low‐grade systemic inflammation and elevated levels of pro‐inflammatory cytokines including tumor necrosis factor‐α (TNF‐α), C‐reactive protein (CRP), interleukin 6 (IL‐6), and IL‐1ß, which could be positively affected by PAP therapy [6, 7, 8]. In addition, markers of endothelial activation such as endothelin‐selectin (E‐selectin) and angiogenic factors including vascular endothelial growth factor (VEGF) and angiopoietin‐2 (Ang2) are elevated in OSA and have been implicated in vascular injury and atherosclerosis. These inflammatory and endothelial biomarkers have demonstrated value as markers of disease severity and mechanistic mediators linking OSA to heightened cardiometabolic risk [9, 10, 11, 12, 13, 14, 15, 16].

While PAP reduces respiratory events and may partially lower systemic inflammation, the evidence has been inconsistent [15, 17, 18, 19, 20, 21, 22, 23]. These findings raise the possibility that CPAP alone does not fully reverse the inflammatory burden of OSA and may leave residual cardiometabolic risk despite effective airway stabilization [9, 10, 24, 25]. To date, the influence of HNS therapy on inflammation in OSA is largely unknown. Our group has previously investigated changes in immune processes relevant to inflammation within the context of OSA therapy. OSAS‐driven oxidative stress is closely associated with the transformation of macrophages into foam cells and strong redistributions of circulating monocyte subsets as a major source of pro‐inflammatory cytokines [26, 27, 28, 29]. Subsequent studies revealed a reconstitution of monocyte subset abundances in OSA patients in response to PAP and HNS therapy, whereas patients with initially more severe monocyte subset alterations showed the most pronounced improvement [30, 31]. Building upon our previous work, this study investigates the effect of HNS therapy on plasma concentrations of CRP, IL‐6, VEGF, TNFα, E‐selectin, and Ang2 in OSA patients. Because systemic inflammation and endothelial dysfunction are pathways connecting OSA to cardiometabolic disease, our findings may offer preliminary insights into the broader role of HNS in modifying cardiometabolic risk.

## 2 Materials and Methods

### 2.1 Ethics Statement

Patients with OSA were selected, implanted and examined during follow‐up at the Department of Otorhinolaryngology, University Hospital Schleswig‐Holstein, Campus Luebeck. All patients provided written informed consent and approval by the ethics committee of the University of Luebeck (approval number 21‐183). Candidates were selected for HNS based on a history of PAP failure, with a BMI (body‐mass‐index) below 35 kg/m^2^, an AHI (apnea‐hypopnea‐index) between 15 and 65 events per hour, and absence of complete concentric collapse at the soft palate during a drug‐induced sleep endoscopy.

### 2.2 Blood Collection and Clinical Data

Blood samples were obtained from healthy donors and OSA patients before HNS implantation and after 6 to 8 months after surgical treatment. Patients' devices were typically activated four to 6 weeks after surgery. AHI, ODI, and time with oxygen saturation below 90% in total sleep time (T90) were recorded via home sleep tests before and after HNS therapy. Additionally, BMI and daytime sleepiness of OSA patients were evaluated before and after HNS treatment using the Epworth Sleepiness Scale (ESS) [32, 33, 34]. Therapy usage was calculated via telemetry read‐out as use per day.

### 2.3 Cytokine Analysis

Plasma concentrations of IL‐6, VEGF, TNF‐α, angiogenic factor angiopoietin 2 (Ang2), acute phase protein C‐reactive protein (CRP), and adhesion molecule E‐selectin were determined by enzyme‐linked immunosorbent assays (ELISA) according to manufacturer's protocols (R&D Systems, Minneapolis, MN, USA).

### 2.4 Statistical Analysis

Statistical analyses were conducted using GraphPad Prism Version 7.0f (GraphPad Software Inc., San Diego, CA, USA). Paired student's *t*‐tests were conducted for pairwise comparison of data before and after HNS treatment. *t*‐Tests for independent samples were conducted to compare data between the healthy controls and OSA patients. The correlation between different clinical or cellular parameters was calculated using multivariate regression with Pearson correlation coefficient. Statistical significance was defined as *p* < 0.05 (*), *p* < 0.01 (**), and *p* < 0.001 (***).

## 3 Results

### 3.1 Patients Characteristics and Clinical Data Following HNS Therapy

Cytokine measurements were performed in plasma samples from 7 healthy donors (3 female/4 male) with a median age of 37.8 years and BMI of 22.7, as well as 18 patients with OSA (5 female/13 male) with a median age of 56.8 years before HNS implantation, as well as after 6 to 8 months HNS therapy (Table 1). Average HNS usage time, assessed by telemetry read‐out from the implant, was 54.1 h/week.

**TABLE 1:** Clinical parameters of the analyzed OSA patients (*n* = 18) as median BMI (body‐mass‐index; kg/m^2^), AHI (apnea‐hypopnea‐index; events/h), ODI (oxygen‐desaturation‐index; events/h), Epworth Sleepiness Scale (ESS) values, and time with oxygen saturation below 90% in total sleep time (T90) before and after HNS therapy, respectively.

	Pre therapy	Post therapy
BMI	28.4 (± 3.5)	28.9 (± 3.5)
AHI	25.9 (± 13.4)	14.2 (± 10.5)
ODI	15.5 (± 11.3)	12.5 (± 8.5)
ESS	12.6 (± 5.0)	7.1 (± 4.0)
T90	11.7 (± 23.8)	6.2 (± 7.9)

*Note:* Values are presented as mean ± SD. AHI per hour less than or equal to 5.0 defines as normal. According to WHO standards, overweight defines as a BMI greater than or equal to 25; ESS less than 10 defines as normal.

**FIGURE 1:**
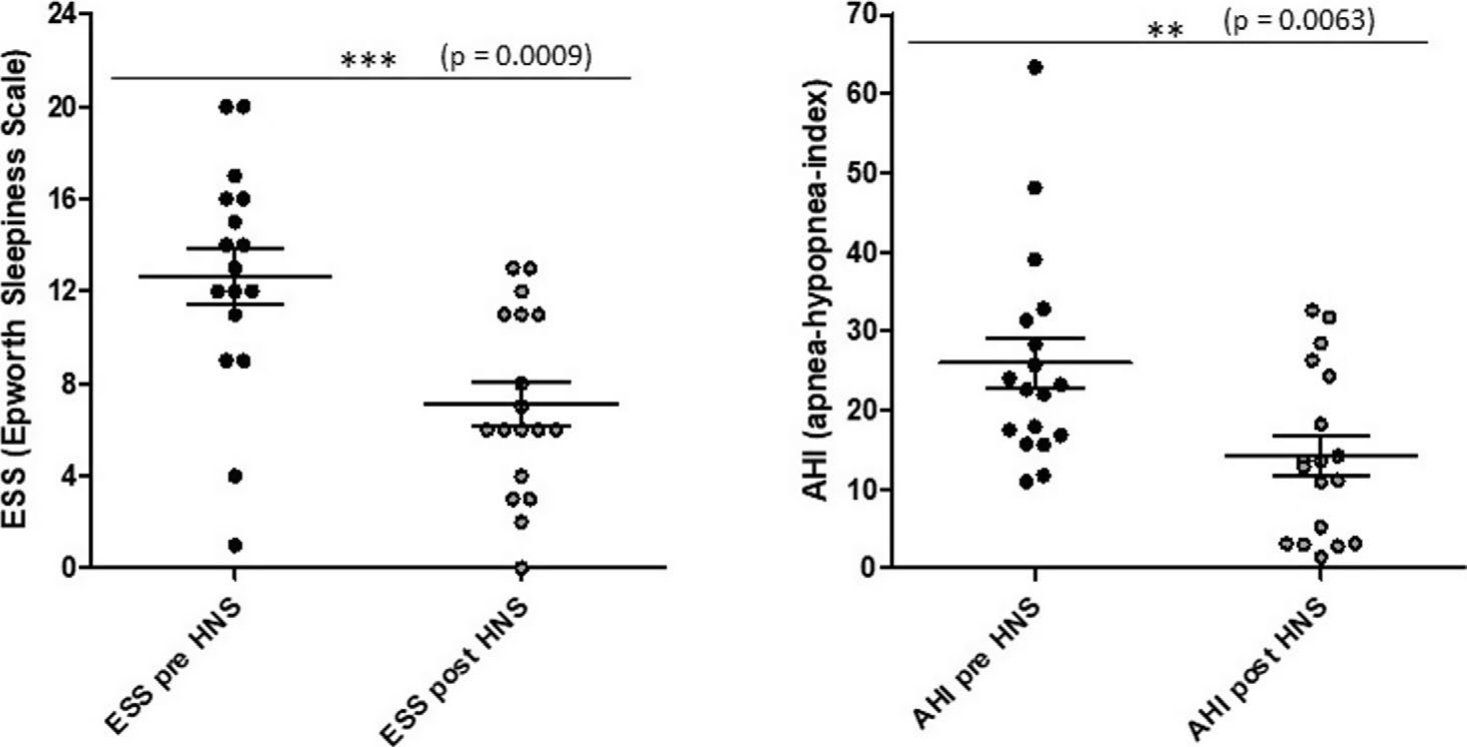
ESS and AHI values in OSA patients upon HNS treatment. Fourteen patients had moderate (AHI 15–30/h) and four had severe OSA (AHI ≥ 30/h). The examined OSA patient cohort revealed overall significantly improved AHI values (apnea‐hypopnea‐index). Initial baseline mean ESS score in OSA group was 12.6, indicating mild excessive daytime sleepiness, which significantly improved upon HNS treatment. **p < 0.01; ***p < 0.001.

The baseline mean ESS score in the OSA group was 12.6, indicating mild excessive daytime sleepiness. Fourteen patients had moderate (AHI 15–30/h) and four had severe OSA (AHI ≥ 30/h). Two patients had normal weight (BMI 20–24.9 kg/m^2^), while 11 patients were overweight (BMI 25–29.9 kg/m^2^) and 5 had class I obesity (BMI 30–34.9 kg/m^2^). In 16 patients, there was no change of more than one point in BMI following HNS treatment, whereas 2 patients had an increase of almost 3 BMI points after 6 months of HNS therapy. AHI and ESS scores were significantly reduced post‐HNS therapy (Figure 1).

### 3.2 Plasma Pro‐Inflammatory Mediators Following HNS Therapy

**FIGURE 2:**
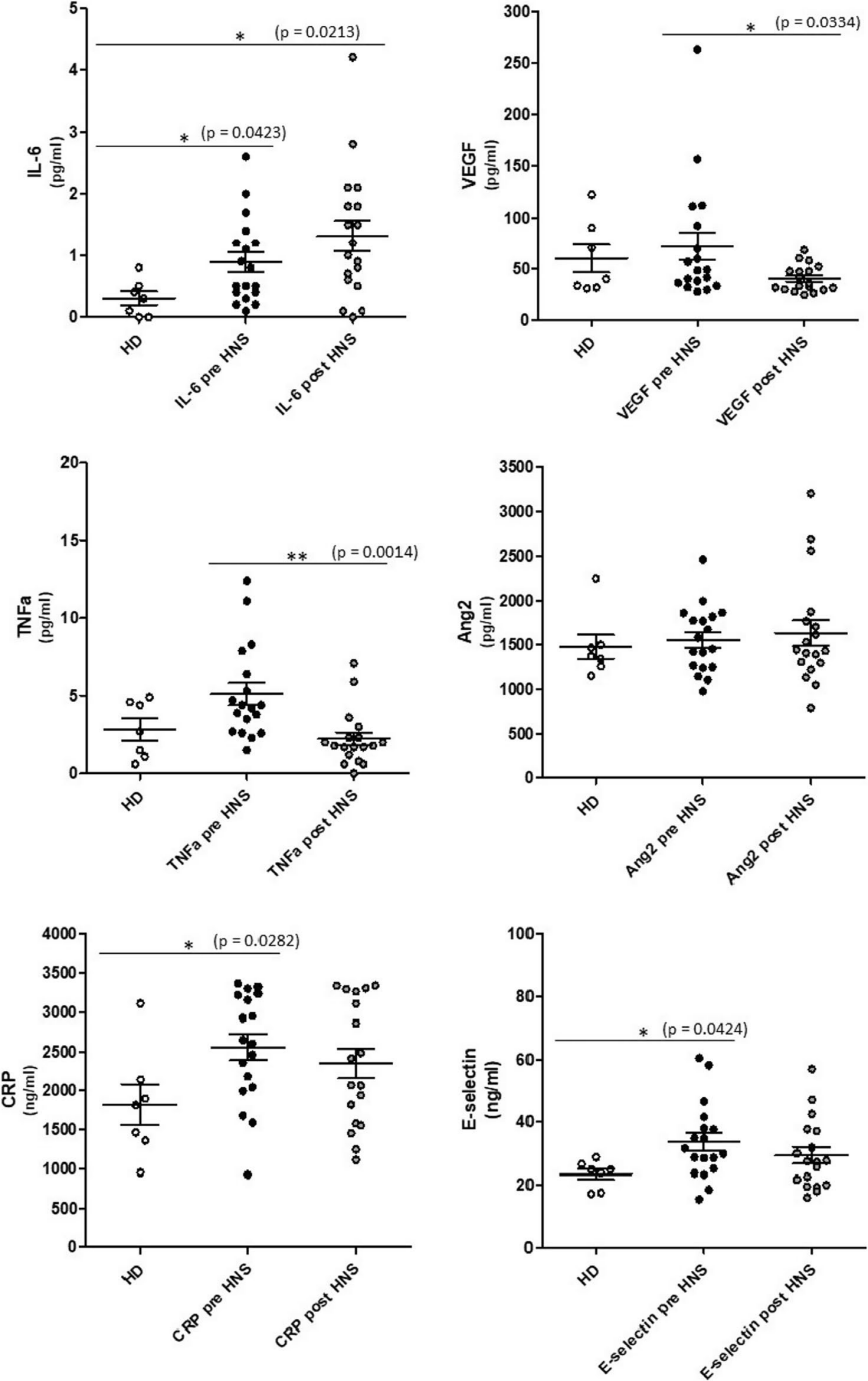
Plasma concentrations of pro‐inflammatory mediators. ELISA measurements of plasma cytokines interleukin 6 (IL‐6), vascular endothelial growth factor (VEGF), tumor necrosis factor‐α (TNF‐α), angiogenic factor angiopoietin 2 (Ang2), acute phase protein C‐reactive protein (CRP), and adhesion molecule E‐selectin in healthy donors (HD) and OSA patients pre versus post HNS therapy. Compared with healthy donors, untreated OSA patients had greater plasma concentrations of IL‐6, VEGF, TNF‐α, CRP, and E‐selectin, whereas Ang2 levels were similar. *p < 0.05; **p < 0.01.

Compared with healthy donors, untreated OSA patients had greater plasma concentrations of IL‐6, VEGF, TNF‐α, CRP, and E‐selectin, whereas Ang2 levels were similar. Consistent with literature [35], IL‐6 and TNF‐α levels were low in both the healthy controls and untreated patients with OSA. HNS therapy was associated with significant reductions in VEGF (*p* = 0.033) and TNF‐α levels (*p* = 0.0013). Levels of CRP, Ang‐2 and E‐selectin remained unchanged after 6 months of HNS therapy (Figure 2). Baseline levels of inflammatory markers were not associated with the severity of OSA as assessed by AHI, ODI, T90, or ESS.

**FIGURE 3:**
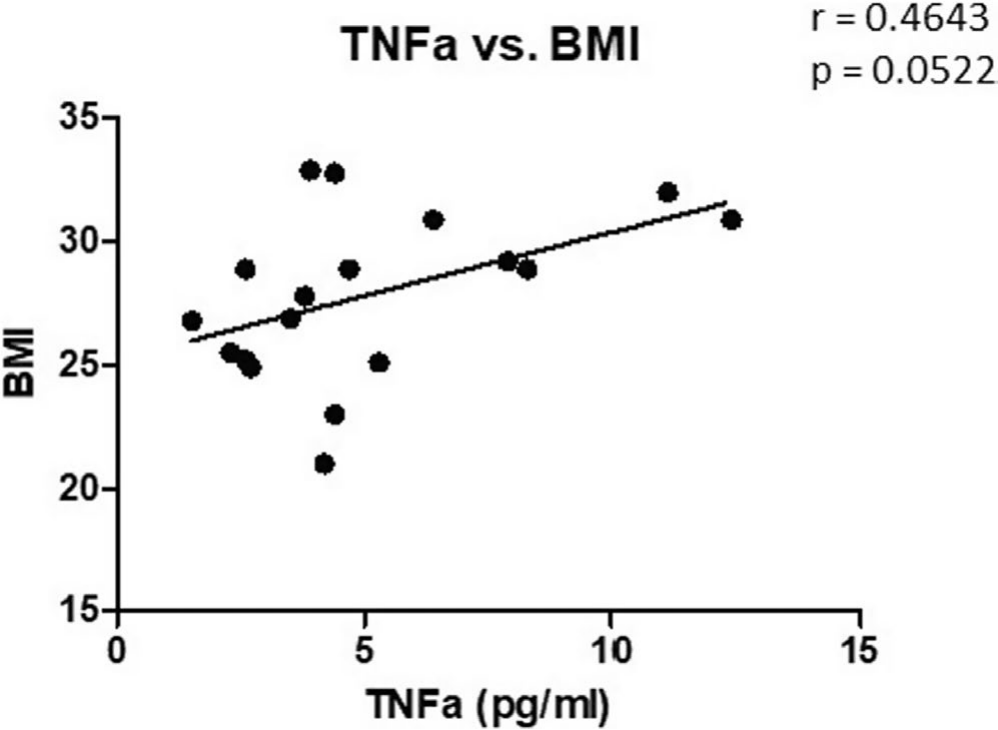
The correlation between different clinical or molecular parameters was calculated using multivariate regression with Pearson correlation coefficient. Data revealed that the association of TNF‐α levels with patients' BMI values was borderline significant. The correlation coefficient (*r*) and *p*‐values are given for each correlation. *p* < 0.05 was considered as significant.

The association between TNF‐α levels and BMI was borderline significant (*p* = 0.0522) (Figure 3).

**FIGURE 4:**
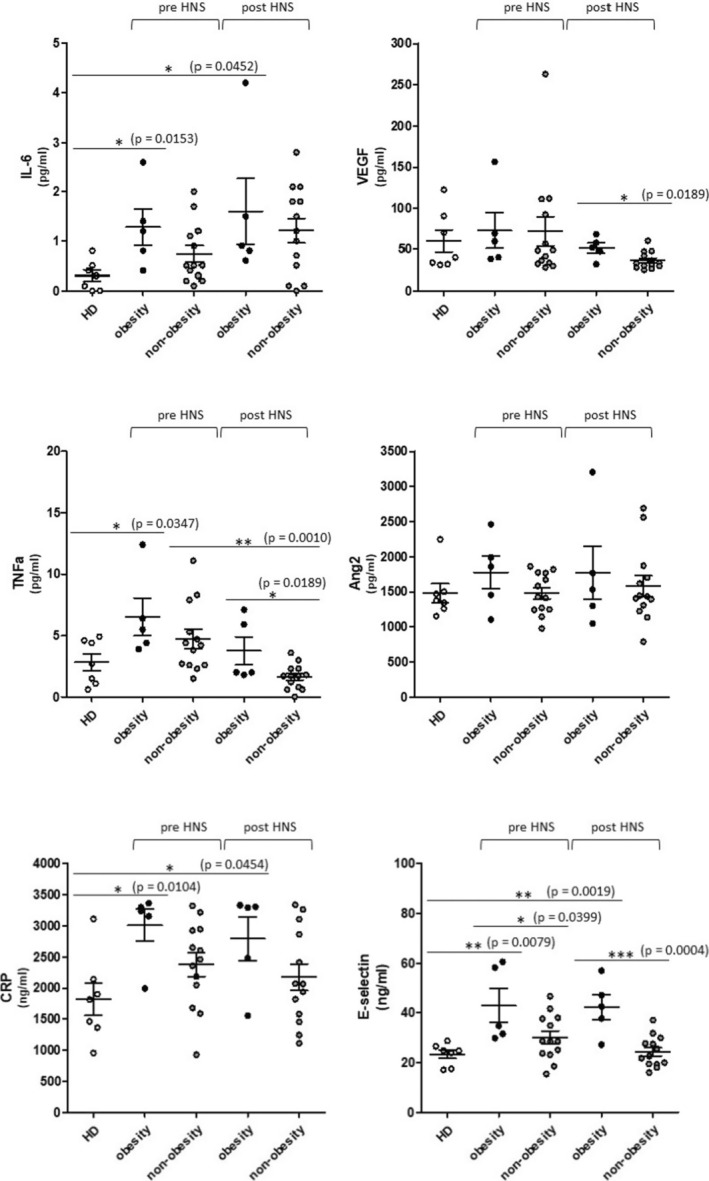
Inflammatory mediators in obesity and non‐obesity subgroups of OSA patients *pre* and *post HNS*. ELISA measurements of plasma cytokines interleukin 6 (IL‐6), vascular endothelial growth factor (VEGF), tumor necrosis factor‐α (TNF‐α), angiogenic factor angiopoietin 2 (Ang2), acute phase protein C‐reactive protein (CRP), and adhesion molecule E‐selectin in healthy donors (HD) and OSA patients with and without obesity. Data revealed that treatment effects differed by subgroup for VEGF and TNF‐α levels. VEGF levels declined significantly in non‐obese patients but not in obese patients while TNF‐α levels declined in obese patients after HNS. ns, not significant. *p < 0.05; **p < 0.01; ***p < 0.001.

To assess the effects of obesity on inflammatory mediators, we stratified patients with OSA into those with obesity (*n* = 5) versus those without obesity (*n* = 13) before HNS therapy (Figure 4). At baseline, obese OSA patients exhibited higher levels of IL‐6, TNF‐α, CRP, and E‐selectin than healthy donors or non‐obese patients with OSA. After HNS, differences by obesity status persisted for IL‐6, CRP, and E‐selectin (Figure 4). Of note, treatment effects differed by subgroup for VEGF and TNF‐α levels. VEGF levels declined significantly in non‐obese patients but not in obese patients, while TNF‐α levels declined in obese patients after HNS (Figure 4).

## 4 Discussion

To our knowledge, this is the first study to examine changes in systemic inflammatory markers with HNS therapy. The major finding of this study is that HNS therapy was associated with partial improvement in select inflammatory markers in patients with OSA. Levels of the angiogenic mediator VEGF and the pro‐inflammatory mediator TNF‐α were reduced whereas levels of IL‐6, CRP, and E‐selectin remained unchanged after 6 months of HNS therapy. VEGF and TNF‐α are crucial, interrelated biomarkers in OSA that reflect the systemic inflammation, chronic intermittent hypoxia, and cardiovascular risk associated with this disorder, both of which are generally elevated in patients with moderate‐to‐severe OSA [36]. Given the role of VEGF as an angiogenic mediator induced by chronic intermittent hypoxia, our early findings suggest that HNS mitigates vascular stress and may promote a more stable endothelial environment through alleviating intermittent hypoxia. Indeed, monitoring VEGF levels has been suggested as a clinically important value for improved endothelial function [37]. The decline in TNF‐α with HNS also aligns with prior literature reporting decreases in TNF‐α after PAP therapy. However, considering well‐documented low baseline levels of TNF‐α in patients with OSA that were confirmed in this study, these changes are of uncertain clinical significance.

Levels of IL‐6, CRP, and E‐selectin did not change with HNS therapy in our study. Prior studies reported inconsistent changes in these inflammatory markers with OSA surgery [38, 39]. High sensitivity ELISA measurements revealed that levels of IL‐6 were low in both healthy controls and OSA groups when compared to ranges reported in previous studies in OSA (approximately 2.5–7 pg/mL) [40, 41, 42], which may have limited our ability to detect a difference with HNS. Our findings regarding CRP mirror several prior studies of CPAP and OSA surgery, in which CRP improvements are inconsistent between studies even though meta‐analyses suggest improvements in CRP with OSA therapy [43] and may be confounded by obesity. Similarly, the effects of CPAP on E‐selectin levels have been inconsistent in prior literature [44].

Obese patients with OSA had higher baseline levels of several inflammatory markers, including IL‐6, TNF‐α, CRP, and E‐selectin, compared with non‐obese patients and healthy donors. These preliminary findings are consistent with prior literature demonstrating that obesity amplifies systemic inflammation in OSA [45]. Because obesity and OSA frequently coexist and both contribute to inflammatory dysregulation and cardiovascular disease [46, 47], obesity is an important potential confounder when interpreting biomarker differences and treatment‐related changes.

This study has several limitations due to the modest sample size of the analyzed cohorts, particularly for the control group, which limited statistical power and multiple comparisons. This may also explain why some markers did not differ significantly and why there was no significant correlation between the measured cytokine values and the clinical parameters such as AHI and ESS. Longer‐term studies may also be valuable to better characterize changes in OSAS‐driven inflammation regulation.

In addition, the healthy donors were younger than the OSA cohort, introducing potential confounding. In a recent publication it has been shown that OSA severity peaked in middle‐aged patients and remained high in older adults, while nocturnal hypoxemia and cardiometabolic comorbidities worsened with age, which underlines that OSA is a dynamic disease with distinct clinical aspects across the lifespan [48].

Furthermore, plasma cytokines IL‐6 and TNF‐α were detected at relatively low concentrations and therefore their relevance in OSAS patients should be interpreted with caution.

Of note, the observational design precludes causal conclusions about long‐term changes in systemic inflammation with HNS and therefore data should be understood as preliminary first indications in this field. Our study also has several strengths. To our knowledge, this is one of the first studies to examine changes in circulating inflammatory and endothelial biomarkers in OSA patients treated with HNS. The prospective, paired pre‐ and post‐therapy design allows each patient to serve as their own control, increasing the potential to detect treatment effects even in a small cohort. Biomarker measurements were performed using standardized ELISA assays in a single laboratory, minimizing inter‐assay variability. In addition, our study included a contemporaneous group of healthy donors for comparison, providing context for interpreting baseline differences in cytokine levels. Finally, the observed reductions in VEGF and TNF‐α are biologically plausible and provide preliminary proof‐of‐concept that HNS may attenuate inflammatory pathways in OSA.

## 5 Conclusion

In summary, HNS therapy was shown to influence the dynamics of inflammatory markers in patients with OSA, suggesting that HNS may attenuate inflammation in OSA. Larger‐scale, randomized studies over longer therapy periods are needed to determine whether the improved inflammatory profile noted in this study translates to improved cardiometabolic outcomes in OSA.

## Funding

The authors have nothing to report.

## Ethics Statement

All patients of the present study (approval number 18‐322 from the ethics committee of the University of Luebeck) were examined and treated at the Department of Otorhinolaryngology, University Hospital Schleswig‐Holstein, Campus Luebeck.

## Consent

The written informed consent was received from all participants.

## Conflicts of Interest

Armin Steffen is working as a consultant for Inspire Medical Inc. Yi Cai reports consulting fees from Aerin Medical. The other authors declare no conflicts of interest.

## Data Availability

The data that support the findings of this study are available from the corresponding author upon reasonable request.

## References

[1] J. L. Chang , A. N. Goldberg , J. A. Alt , et al., “International Consensus Statement on Obstructive Sleep Apnea,” International Forum of Allergy & Rhinology 13, no. 7 (2023): 1061–1482. 36068685 10.1002/alr.23079 PMC10359192

[2] V. K. Kapur , D. H. Auckley , S. Chowdhuri , et al., “Clinical Practice Guideline for Diagnostic Testing for Adult Obstructive Sleep Apnea: An American Academy of Sleep Medicine Clinical Practice Guideline,” Journal of Clinical Sleep Medicine 13, no. 3 (2017): 479–504. 28162150 10.5664/jcsm.6506 PMC5337595

[3] A. Maniaci , G. Iannella , S. Cocuzza , et al., “Oxidative Stress and Inflammation Biomarker Expression in Obstructive Sleep Apnea Patients,” Journal of Clinical Medicine 10, no. 2 (2021): 277. 33451164 10.3390/jcm10020277 PMC7828672

[4] P. J. Strollo, Jr. , R. J. Soose , J. T. Maurer , et al., “Upper‐Airway Stimulation for Obstructive Sleep Apnea,” New England Journal of Medicine 370, no. 2 (2014): 139–149. 24401051 10.1056/NEJMoa1308659

[5] A. Steffen , C. Heiser , W. Galetke , et al., “Hypoglossal Nerve Stimulation for Obstructive Sleep Apnea: Updated Position Paper of the German Society of Oto‐Rhino‐Laryngology, Head and Neck Surgery,” European Archives of Oto‐Rhino‐Laryngology 279, no. 1 (2022): 61–66. 34151387 10.1007/s00405-021-06902-6 PMC8738404

[6] F. Jin , J. Liu , X. Zhang , et al., “Effect of Continuous Positive Airway Pressure Therapy on Inflammatory Cytokines and Atherosclerosis in Patients With Obstructive Sleep Apnea Syndrome,” Molecular Medicine Reports 16, no. 5 (2017): 6334–6339. 28901415 10.3892/mmr.2017.7399

[7] P. Steiropoulos , I. Kotsianidis , E. Nena , et al., “Long‐Term Effect of Continuous Positive Airway Pressure Therapy on Inflammation Markers of Patients With Obstructive Sleep Apnea Syndrome,” Sleep 32, no. 4 (2009): 537–543. 19413148 10.1093/sleep/32.4.537 PMC2663658

[8] I. Bouloukaki , C. Mermigkis , E. Kallergis , V. Moniaki , E. Mauroudi , and S. Schiza , “Obstructive Sleep Apnea Syndrome and Cardiovascular Disease: The Influence of C‐Reactive Protein,” World Journal of Experimental Medicine 5, no. 2 (2015): 77–83. 25992322 10.5493/wjem.v5.i2.77 PMC4436942

[9] Y. Peker , Y. Celik , A. Behboudi , et al., “CPAP May Promote an Endothelial Inflammatory Milieu in Sleep Apnoea After Coronary Revascularization,” eBioMedicine 101 (2024): 105015. 38403558 10.1016/j.ebiom.2024.105015 PMC10944158

[10] D. J. Gottlieb , S. L. Hansen , R. L. McClelland , et al., “Association of Obstructive Sleep Apnea With Soluble Biomarkers of Lung and Vascular Injury: The Multi‐Ethnic Study of Atherosclerosis,” Sleep 48, no. 4 (2025): zsae271. 39565921 10.1093/sleep/zsae271 PMC11985382

[11] O. Cohen , V. Kundel , F. Barbe , et al., “The Great Controversy of Obstructive Sleep Apnea Treatment for Cardiovascular Risk Benefit: Advancing the Science Through Expert Consensus. An Official American Thoracic Society Workshop Report,” Annals of the American Thoracic Society 22, no. 1 (2024): 1–22. 10.1513/AnnalsATS.202409-981ST PMC11708754 39513996

[12] B. U. Peres , A. J. Hirsch Allen , P. Daniele , et al., “Circulating Levels of Cell Adhesion Molecules and Risk of Cardiovascular Events in Obstructive Sleep Apnea,” PLoS One 16, no. 7 (2021): e0255306. 34329349 10.1371/journal.pone.0255306 PMC8323915

[13] K. L. J. Cederberg , U. Hanif , V. Peris Sempere , et al., “Proteomic Biomarkers of the Apnea Hypopnea Index and Obstructive Sleep Apnea: Insights Into the Pathophysiology of Presence, Severity, and Treatment Response,” International Journal of Molecular Sciences 23, no. 14 (2022): 7983. 35887329 10.3390/ijms23147983 PMC9317550

[14] F. Campos‐Rodriguez , M. I. Asensio‐Cruz , J. Cordero‐Guevara , et al., “Effect of Continuous Positive Airway Pressure on Inflammatory, Antioxidant, and Depression Biomarkers in Women With Obstructive Sleep Apnea: A Randomized Controlled Trial,” Sleep 42, no. 10 (2019): zsz145. 31314107 10.1093/sleep/zsz145

[15] E. Thunstrom , H. Glantz , T. Yucel‐Lindberg , K. Lindberg , M. Saygin , and Y. Peker , “CPAP Does Not Reduce Inflammatory Biomarkers in Patients With Coronary Artery Disease and Nonsleepy Obstructive Sleep Apnea: A Randomized Controlled Trial,” Sleep 40, no. 11 (2017): zsx157. 10.1093/sleep/zsx157 29029237

[16] R. Lorbeer , S. E. Baumeister , M. Dorr , et al., “Circulating Angiopoietin‐2, Its Soluble Receptor Tie‐2, and Mortality in the General Population,” European Journal of Heart Failure 15, no. 12 (2013): 1327–1334. 23901057 10.1093/eurjhf/hft117

[17] X. Xie , L. Pan , D. Ren , C. Du , and Y. Guo , “Effects of Continuous Positive Airway Pressure Therapy on Systemic Inflammation in Obstructive Sleep Apnea: A Meta‐Analysis,” Sleep Medicine 14, no. 11 (2013): 1139–1150. 24054505 10.1016/j.sleep.2013.07.006

[18] A. Baessler , R. Nadeem , M. Harvey , et al., “Treatment for Sleep Apnea by Continuous Positive Airway Pressure Improves Levels of Inflammatory Markers ‐ a Meta‐Analysis,” Journal of Inflammation 10 (2013): 13. 23518041 10.1186/1476-9255-10-13 PMC3637233

[19] Y. Guo , L. Pan , D. Ren , and X. Xie , “Impact of Continuous Positive Airway Pressure on C‐Reactive Protein in Patients With Obstructive Sleep Apnea: A Meta‐Analysis,” Sleep 17, no. 2 (2013): 495–503. 10.1007/s11325-012-0722-2 22664769

[20] R. B. Kutler , I. F. Caplan , S. Jelic , M. P. St‐Onge , and Y. Cai , “The Metabolic Outcomes of Obstructive Sleep Apnea Surgery: A Scoping Review,” Laryngoscope 135 (2025): 3508–3519. 40353761 10.1002/lary.32255

[21] M. Friedman , C. G. Samuelson , C. Hamilton , et al., “Effect of Continuous Positive Airway Pressure on C‐Reactive Protein Levels in Sleep Apnea: A Meta‐Analysis,” Otolaryngology and Head and Neck Surgery 147, no. 3 (2012): 423–433. 10.1177/0194599812451840 22714423

[22] M. Kohler , L. Ayers , J. C. Pepperell , et al., “Effects of Continuous Positive Airway Pressure on Systemic Inflammation in Patients With Moderate to Severe Obstructive Sleep Apnoea: A Randomised Controlled Trial,” Thorax 64, no. 1 (2009): 67–73. 18786982 10.1136/thx.2008.097931

[23] Q. Zhu , Q. Luo , Z. Wang , S. Chen , G. Chen , and S. Huang , “Effects of Continuous Positive Airway Pressure Therapy on Inflammatory Markers in Patients With Obstructive Sleep Apnea: A Meta‐Analysis of Randomized Controlled Trials,” Sleep 29, no. 2 (2025): 182. 10.1007/s11325-025-03348-6 PMC12064446 40346316

[24] R. Shah , N. Patel , M. Emin , et al., “Statins Restore Endothelial Protection Against Complement Activity in Obstructive Sleep Apnea: A Randomized Clinical Trial,” Annals of the American Thoracic Society 20, no. 7 (2023): 1029–1037. 36912897 10.1513/AnnalsATS.202209-761OC PMC12039953

[25] D. J. Gottlieb , D. J. Lederer , J. S. Kim , et al., “Effect of Positive Airway Pressure Therapy of Obstructive Sleep Apnea on Circulating Angiopoietin‐2,” Sleep Medicine 96 (2022): 119–121. 35636149 10.1016/j.sleep.2022.05.007 PMC9813950

[26] S. Tamaki , M. Yamauchi , A. Fukuoka , et al., “Production of Inflammatory Mediators by Monocytes in Patients With Obstructive Sleep Apnea Syndrome,” Internal Medicine 48, no. 15 (2009): 1255–1262. 19652426 10.2169/internalmedicine.48.2366

[27] H. Inonu Koseoglu , A. C. Pazarli , A. Kanbay , and O. Demir , “Monocyte Count/HDL Cholesterol Ratio and Cardiovascular Disease in Patients With Obstructive Sleep Apnea Syndrome: A Multicenter Study,” Clinical and Applied Thrombosis/Hemostasis 24, no. 1 (2018): 139–144. 27837155 10.1177/1076029616677803 PMC6714621

[28] J. Zhou , W. Bai , Q. Liu , J. Cui , and W. Zhang , “Intermittent Hypoxia Enhances THP‐1 Monocyte Adhesion and Chemotaxis and Promotes M1 Macrophage Polarization via RAGE,” BioMed Research International 2018 (2018): 1650456. 30402462 10.1155/2018/1650456 PMC6196992

[29] C. Polasky , A. Steffen , K. Loyal , C. Lange , K. Bruchhage , and R. Pries , “Redistribution of Monocyte Subsets in Obstructive Sleep Apnea Syndrome Patients Leads to an Imbalanced PD‐1/PD‐L1 Cross‐Talk With CD4/CD8 T Cells,” Journal of Immunology 206, no. 1 (2021): 51–58. 10.4049/jimmunol.2001047 33268482

[30] C. Polasky , A. Steffen , K. Loyal , C. Lange , K. L. Bruchhage , and R. Pries , “Reconstitution of Monocyte Subsets and PD‐L1 Expression but Not T Cell PD‐1 Expression in Obstructive Sleep Apnea Patients Upon PAP Therapy,” International Journal of Molecular Sciences 22, no. 21 (2021): 11375. 34768806 10.3390/ijms222111375 PMC8583745

[31] R. Pries , C. Lange , N. Behn , K. L. Bruchhage , and A. Steffen , “Dynamics of Circulating CD14/CD16 Monocyte Subsets in Obstructive Sleep Apnea Syndrome Patients Upon Hypoglossal Nerve Stimulation,” Biomedicine 10, no. 8 (2022): 1925. 10.3390/biomedicines10081925 PMC9405940 36009474

[32] Y. H. Chen , M. F. Wu , C. Y. Wen , et al., “Interactions Between Obstructive Sleep Apnea Syndrome Severity, Obesity, Sex Difference and Attention‐Deficit/Hyperactivity Disorder on Health‐Related Quality of Life: A Non‐Interventional Prospective Observational Study,” Biomedicine 10, no. 7 (2022): 1576. 10.3390/biomedicines10071576 PMC9313041 35884881

[33] M. W. Johns , “A New Method for Measuring Daytime Sleepiness: The Epworth Sleepiness Scale,” Sleep 14, no. 6 (1991): 540–545. 1798888 10.1093/sleep/14.6.540

[34] S. Lee , P. Song , S. Choi , S. Suh , S. Kwon , and E. Joo , “The Impact of the Shift System on Health and Quality of Life of Sleep Technicians,” Sleep Medicine 76 (2020): 72–79. 33120131 10.1016/j.sleep.2020.09.026

[35] S. Ryan , C. T. Taylor , and W. T. McNicholas , “Systemic Inflammation: A Key Factor in the Pathogenesis of Cardiovascular Complications in Obstructive Sleep Apnoea Syndrome?,” Thorax 64, no. 7 (2009): 631–636. 19561283 10.1136/thx.2008.105577

[36] K. Archontogeorgis , E. Nena , N. Papanas , et al., “Serum Levels of Vascular Endothelial Growth Factor and Insulin‐Like Growth Factor Binding Protein‐3 in Obstructive Sleep Apnea Patients: Effect of Continuous Positive Airway Pressure Treatment,” Open Cardiovascular Medicine Journal 9 (2015): 133–138. 27006717 10.2174/1874192401509010133 PMC4768659

[37] J. C. Qi , L. Zhang , H. Li , et al., “Impact of Continuous Positive Airway Pressure on Vascular Endothelial Growth Factor in Patients With Obstructive Sleep Apnea: A Meta‐Analysis,” Sleep 23, no. 1 (2019): 5–12. 10.1007/s11325-018-1660-4 29671205

[38] F. Wang , Y. Liu , H. Xu , et al., “Association Between Upper‐Airway Surgery and Ameliorative Risk Markers of Endothelial Function in Obstructive Sleep Apnea,” Scientific Reports 9, no. 1 (2019): 20157. 31882827 10.1038/s41598-019-56601-w PMC6934655

[39] B. S. Y. Yeo , J. H. Koh , B. K. J. Tan , et al., “Improved Inflammatory and Cardiometabolic Profile After Soft‐Tissue Sleep Surgery for Obstructive Sleep Apnea: A Systematic Review and Meta‐Analysis,” JAMA Otolaryngology. Head & Neck Surgery 148, no. 9 (2022): 862–869. 35951318 10.1001/jamaoto.2022.2285 PMC9372898

[40] M. M. Imani , M. Sadeghi , H. Khazaie , M. Emami , D. Sadeghi Bahmani , and S. Brand , “Evaluation of Serum and Plasma Interleukin‐6 Levels in Obstructive Sleep Apnea Syndrome: A Meta‐Analysis and Meta‐Regression,” Frontiers in Immunology 11 (2020): 1343. 32793188 10.3389/fimmu.2020.01343 PMC7385225

[41] S. O. Wali , J. Al‐Mughales , F. Alhejaili , et al., “The Utility of Proinflammatory Markers in Patients With Obstructive Sleep Apnea,” Sleep 25, no. 2 (2021): 545–553. 10.1007/s11325-020-02149-3 32705528

[42] P. Fiedorczuk , E. Olszewska , A. Polecka , M. Walasek , B. Mroczko , and A. Kulczynska‐Przybik , “Investigating the Role of Serum and Plasma IL‐6, IL‐8, IL‐10, TNF‐Alpha, CRP, and S100B Concentrations in Obstructive Sleep Apnea Diagnosis,” International Journal of Molecular Sciences 24, no. 18 (2023): 13875. 37762178 10.3390/ijms241813875 PMC10530258

[43] C. H. Lee , W. C. Hsu , T. H. Yeh , J. Y. Ko , M. T. Lin , and K. T. Kang , “Effect of Sleep Surgery on Inflammatory Cytokines in Adult Obstructive Sleep Apnea: A Systematic Review and Meta‐Analysis,” Laryngoscope 132, no. 11 (2022): 2275–2284. 35567416 10.1002/lary.30176

[44] Z. Tian , J. Xiao , J. Kang , et al., “Effects of Continuous Positive Airway Pressure on Cell Adhesion Molecules in Patients With Obstructive Sleep Apnea: A Meta‐Analysis,” Lung 199, no. 6 (2021): 639–651. 34800156 10.1007/s00408-021-00487-x

[45] E. S. Arnardottir , G. Maislin , R. J. Schwab , et al., “The Interaction of Obstructive Sleep Apnea and Obesity on the Inflammatory Markers C‐Reactive Protein and Interleukin‐6: The Icelandic Sleep Apnea Cohort,” Sleep 35, no. 7 (2012): 921–932. 22754038 10.5665/sleep.1952 PMC3369227

[46] C. Poitou , E. Dalmas , M. Renovato , et al., “CD14dimCD16+ and CD14+CD16+ Monocytes in Obesity and During Weight Loss: Relationships With Fat Mass and Subclinical Atherosclerosis,” Arteriosclerosis, Thrombosis, and Vascular Biology 31, no. 10 (2011): 2322–2330. 21799175 10.1161/ATVBAHA.111.230979

[47] J. Gaines , A. N. Vgontzas , J. Fernandez‐Mendoza , et al., “Inflammation Mediates the Association Between Visceral Adiposity and Obstructive Sleep Apnea in Adolescents,” American Journal of Physiology Endocrinology and Metabolism 311, no. 5 (2016): E851–E858. 27651112 10.1152/ajpendo.00249.2016 PMC5130357

[48] A. Tzinas , A. Karkala , S. C. Kotoulas , et al., “Age and Sex Matter: Phenotypic Heterogeneity, Diagnostic Gaps, and Screening Tool Performance in Obstructive Sleep Apnea‐A 10‐Year Sleep Clinic Cohort Study,” Diagnostics 16, no. 7 (2026): 983. 41975696 10.3390/diagnostics16070983 PMC13073577

